# Supplementary material for: Orange maker: a CRISPR/Cas9-mediated genome editing and screening project to generate orange-eyed DarkJedi GAL4 lines by undergraduate students
Source: Sci Rep. 2024 Aug 13;14:18778. doi: 10.1038/s41598-024-69946-8 (PMC11322649; doi:10.1038/s41598-024-69946-8)
Supplement: Supplementary file 1 — Supplementary Figures. [file 41598_2024_69946_MOESM1_ESM.pdf]

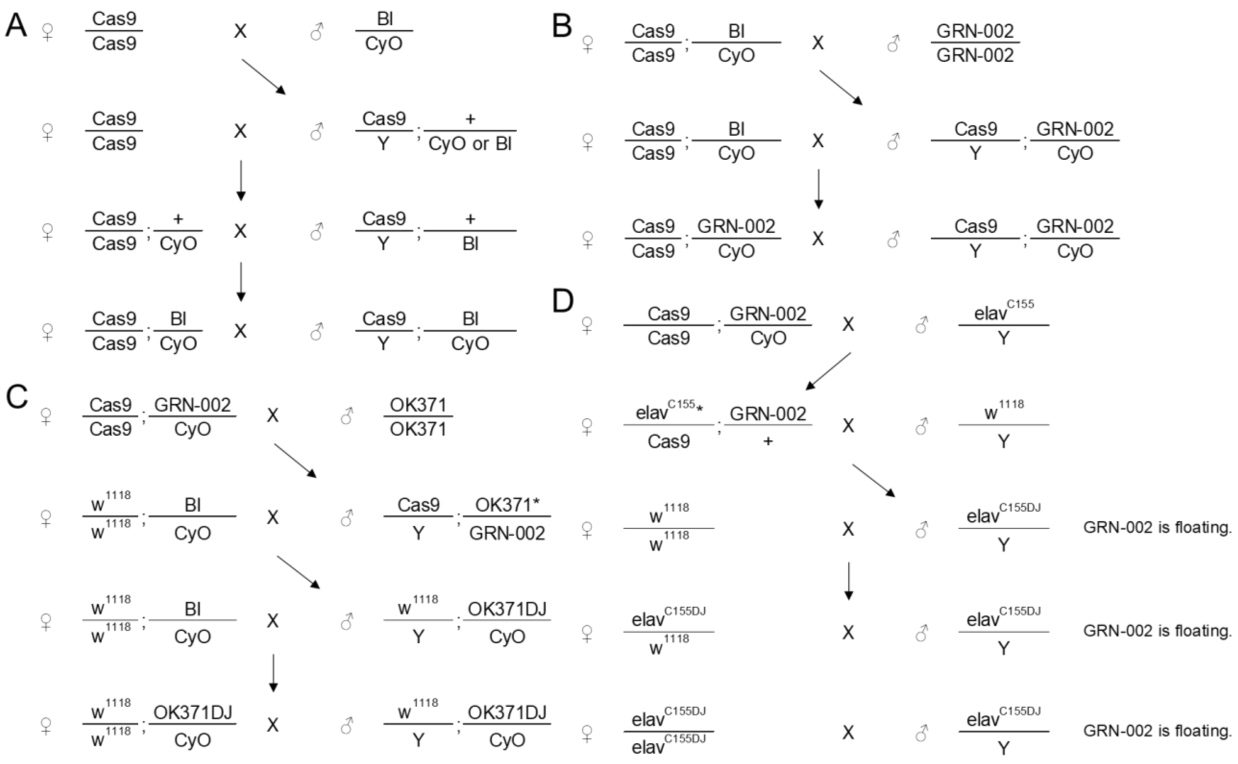

Supplementary Figure 1. Mating schemes for the generation of the Orange Maker and orange making with the Orange Maker. (A) Balanced Cas9 generation, (B) Orange Maker generation, (C) Orange making (DarkJedi line generation) with OK371, (D) Orange making (DarkJedi line generation) with elav^{C155}-GAL4**,** see the method section for a detailed explanation. *: modified (mosaic) GAL4 lines by maternally deposited active Cas9-gRNA complex, DJ: selected orange-eyed fly

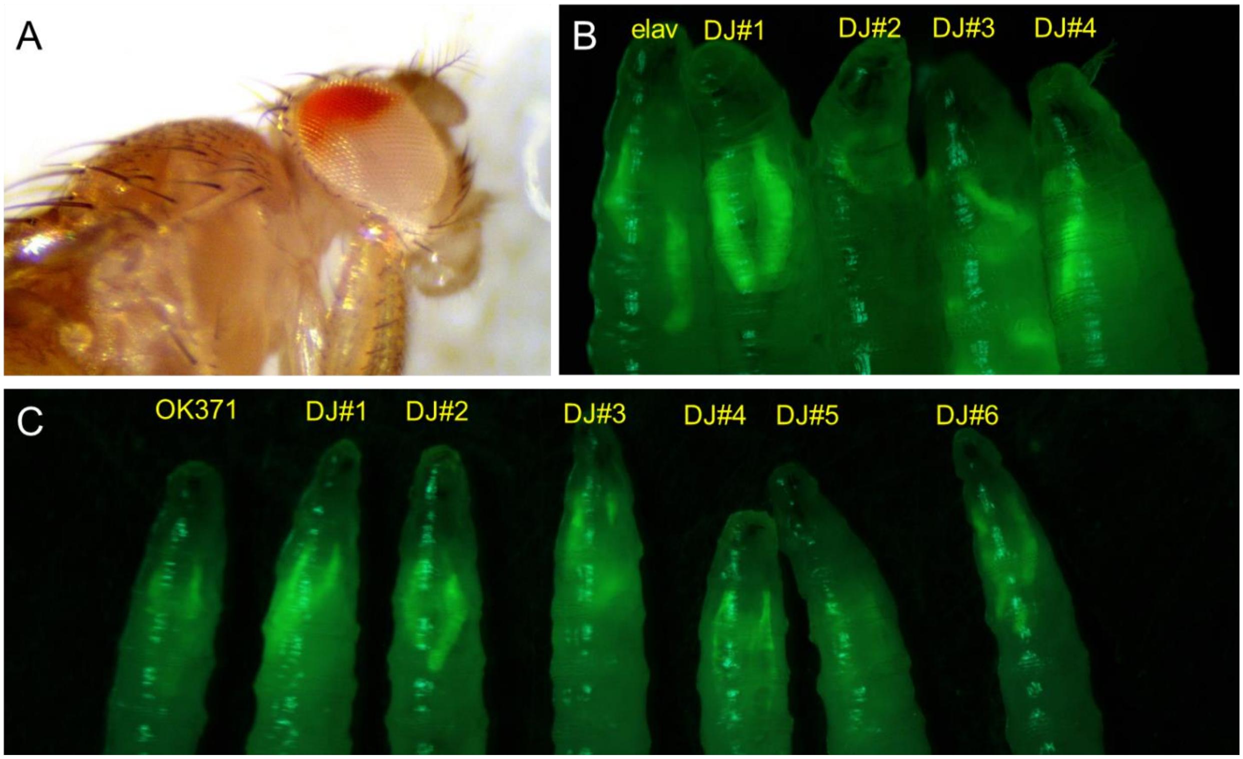

Supplementary Figure 2. (A) An example of the mosaic-eyed fly (Cas9/Y; OK371*/GRN-002 in Fig. S1C)**,** (B) GFP expression derived by elav^{C155DJ} lines in intact larvae with the DIY fluorescence stereoscope setup, (C) GFP expression derived by OK371^{DJ} lines in intact larvae with the DIY fluorescence stereoscope setup. Preliminary assessments for the activity of DarkJedi lines were possible by observing the strong salivary glands GFP signals along with relative weak signals in the central nervous system through the cuticle without careful dissection.
